# Recent Advances on the Development of Protein-Based Adhesives for Wood Composite Materials—A Review

**DOI:** 10.3390/molecules26247617

**Published:** 2021-12-15

**Authors:** Nidal Del Valle Raydan, Leo Leroyer, Bertrand Charrier, Eduardo Robles

**Affiliations:** University of Pau and the Adour Region, E2S UPPA, CNRS, IPREM-UMR 5254, 40004 Mont de Marsan, France; ndvraydan@univ-pau.fr (N.D.V.R.); leo.leroyer@univ-pau.fr (L.L.); bertrand.charrier@univ-pau.fr (B.C.)

**Keywords:** biobased adhesives, plant proteins, animal proteins, adhesion theories, wood adhesives

## Abstract

The industrial market depends intensely on wood-based composites for buildings, furniture, and construction, involving significant developments in wood glues since 80% of wood-based products use adhesives. Although biobased glues have been used for many years, notably proteins, they were replaced by synthetic ones at the beginning of the 20th century, mainly due to their better moisture resistance. Currently, most wood adhesives are based on petroleum-derived products, especially formaldehyde resins commonly used in the particleboard industry due to their high adhesive performance. However, formaldehyde has been subjected to strong regulation, and projections aim for further restrictions within wood-based panels from the European market, due to its harmful emissions. From this perspective, concerns about environmental footprint and the toxicity of these formulations have prompted researchers to re-investigate the utilization of biobased materials to formulate safer alternatives. In this regard, proteins have sparked a new and growing interest in the potential development of industrial adhesives for wood due to their advantages, such as lower toxicity, renewable sourcing, and reduced environmental footprint. This work presents the recent developments in the use of proteins to formulate new wood adhesives. Herein, it includes the historical development of wood adhesives, adhesion mechanism, and the current hotspots and recent progress of potential proteinaceous feedstock resources for adhesive preparation.

## 1. Introduction

Wood bonding has been practiced for many ages. Bonding or gluing of solid wood, wood particles of different shapes and sizes, and wood fibers is needed to produce elaborate wood-based products, whether for use in construction, furniture, or other applications. The first adhesives used for the wood industry were made from natural resources such as collagen, blood, casein, fish, starch, and other derivatives in the 19th and 20th centuries. However, the production of bioadhesives dropped dramatically due to the boom of products made from fossil sources starting in the 1930s [1]. These synthetic adhesives replaced renewable ones as a result of their low cost and availability, but also due to their superior strength and resistance to moisture.

Consequently, most of the industrially used wood adhesives are made from fossil-derived polymers; conventional adhesives are contingent on four major synthetic thermosetting resins: PF (-alkaline catalyst salt), UF (-acidic catalyst salt), MF, and polymeric diphenylmethane diisocyanate (pMDI) resin [2,3,4,5,6]. Currently, the wood panel industry uses almost 95% of synthetic petroleum-derived thermosetting adhesives, mainly based on urea, phenol, and melamine, among others [7,8,9,10]. For example, pressed wood products, such as wood-based particleboard and medium density fiberboards (MDF), and oriented strand board (OSB) are made using UF-based resins [9,11,12]. UF-based adhesives are almost exclusively used for producing wood-based materials, such as particleboard or medium-density fiberboards (MDF) for interior applications [13], due to their low-cost raw materials, their rapid curing, their high dry bond strength, and a colorless glue line, However, most of the adhesive resins containing urea-formaldehyde cause health concerns during production and service, due to residual unreacted formaldehyde and slow adhesive hydrolysis under hot/humid conditions. Off-gassing levels are highest when the products are new, with emissions tapering off as the products get older. Exposure to free formaldehyde in concentrations greater than 0.1 parts per million (ppm) can cause nasal and throat congestions, burning eyes, respiratory tract irritation, as well as skin sensitization [14,15]. Moreover, prolonged exposure to a high-dose of free formaldehyde increases the risk of causing cancers. Accordingly, the International Agency for Research on Cancer (IARC) classifies formaldehyde as a group 1 carcinogenic substance [16,17]. Therefore, growing emphasis on green and sustainable buildings and emission regulations regarding the chemicals used for processing or manufacturing wood or wood composites for home applications are significant driving factors to limit formaldehyde-based resins [18,19,20,21,22,23,24].

Moreover, formaldehyde has been recorded under REACH as a hazardous and toxic compound, and its use may be restricted and substituted with less toxic adhesives (European Commission) [13]. These disadvantages induced a favorable scenario for researching and developing a new generation of adhesives obtained from biobased sources without generating free formaldehyde or other volatile organic compound (VOC) emissions [25], although many ways are being used to reduce free formaldehyde emissions, for instance, by adding formaldehyde scavengers to conventional wood adhesives, surface treatment (of wood composites), or using eco-friendly adhesive formulations [25,26,27,28]. However, another aspect promoting the use of such products is the volatility and the political dependence of oil prices; in addition, renewable raw materials or waste biomass contributes to reducing the carbon footprint and constitutes a path towards the zero-carbon goals [29].

There are several reports of different biomass resources to produce biobased adhesives [30]. Among the various biopolymers, proteins are an abundant class of macromolecules since they function as the main organic building blocks of living organisms, composing about 50 percent of the dry cell weight [31]. Therefore, there has been an increased interest in using proteins from plant and animal sources for applications such as glues and adhesives. However, as their structure and, therefore, their properties are adapted to their original natural environment, chemical modifications may be necessary if they are to be used in different contexts, especially where high performance is required. For example, due to the many polar groups present in proteins, water resistance is the foremost hurdle to overcome [32]. In addition, studying the theories and mechanisms responsible for wood adhesive bonding has generated particular interest over the last century, requiring a better understanding of the characteristics of wood materials and polymers, as well as surface science. Therefore, to illustrate these various points, the current review intends to present the historical evolution of wood adhesives, the mechanics and wood-related considerations behind bioadhesive development, and the recent literature on protein-based wood adhesives from a qualitative perspective.

## 2. History of Wood Adhesives

### 2.1. Evolution of Adhesives: From Natural to Synthetic

Things that we recognize as bonding adhesives today have an ancient history. One of the earliest known adhesives was for hafting tools onto the wood in the Middle Stone Age, for example, with a sticky substance made from tree sap and red ocher in South Africa [33]. From this period, techniques continued to grow; adhesive applications became increasingly used daily for engineering and artistic purposes. Egyptians first introduced liquid animal-based adhesives in the eighteenth-century BCE in their wooden artifacts. The early Greeks and Romans also developed adhesive techniques for veneering and marquetry in their woodwork [34]. The origin of all these adhesives was natural, meaning from plants or animals. Some could be used as glue without pretreatment, like blood, gums, pitch, and rubber latex, unlike soy protein, milk casein, and collagen adhesives, which require more processing. This first generation of wood glues required low strength as they were used for interior applications, such as furniture. The desire to use wood more efficiently was an impetus for adhesive development in the 19th and 20th centuries. Tannins have been used for many years as a wood adhesive in locations where they are readily available and where phenolic compounds are more limited in supply and are costlier. On the other hand, carbohydrates are not widely used in wood bonding due to their water sensitivity.

However, despite some success with biobased adhesives, the turning points in the bioadhesive story were the Industrial Revolution and the appearance of synthetic adhesives starting in the 1930s, mainly due to economics, water resistance, and ease of use. Phenol-formaldehyde (PF) was one of the first synthetic adhesives, and its application to wood bonding in the 1930s permitted the development of durable exterior plywood (PW). Urea-formaldehyde (UF), a low-cost and effective adhesive, led to the growth of existing interior products and new panel products. Melamine-formaldehyde (MF) adhesives are more water-resistant but more expensive than urea-formaldehyde. They have been used to improve urea-formaldehyde adhesives or used themselves as an exterior adhesive, especially outside North America. World War II was a significant period for improving and growing many other synthetic polymers. The most fundamental of which was polyvinyl acetate and polyurethane. Other adhesive products consisted of epoxies, aliphatic resins, and construction adhesives. The main benefit of these adhesives is that they can be formulated to have a wide variety of properties, according to the types and ratios of monomers. In addition, the ability to formulate the polymer backbone rather than using what nature has provided is an essential advantage of synthetics over natural products [35], knowing that adhesion-improving modifications of biobased raw materials is feasible to achieve the desired properties. However, synthetic adhesives present drawbacks such as high free formaldehyde emissions, pollution during production and use, recycling difficulties, and dependence on a controversial resource, namely oil.

### 2.2. Formaldehyde Legislation

Legislation regarding the indoor environment and final product emissions have consistently become stricter over time. The World Health Organization (WHO) has set guidelines for indoor air concentrations of free formaldehyde of 0.1 mg/m^3^ [36]. Non-occupational indoor exposure limits have been placed at the WHO guideline of 0.1 mg/m^3^ by many countries such as the UK, the People’s Republic of China, and Japan. Since the 1980s, some European countries have introduced national regulations on specific products that emit free formaldehyde. For example, formaldehyde emission class E112 (0.1 ppm boards) became obligatory for wood-based panels in Austria, Denmark, Germany, and Sweden in 1985. Emission classes, E1 and E2, were established by European Standard EN 13986 for wood products used in construction in 2004, and in 2006, emission class E1 became effective for panel production [37,38]. The federal-state committee for chemicals decided on stricter legal limit values of free formaldehyde emissions from wood-based materials. The new E05 standard was only applied in Germany on 1 January 2020 [39]. In the US, a critical regulatory development introduced the airborne toxic control measures to reduce free formaldehyde emissions from composite wood products in 2008 by the California Air Resources Board (CARB) [40]. After that, this formed the basis for developing the national “Formaldehyde Standards for Composite Wood Products Act” (2010) for various products, such as PW, MDF, and others. Japan has its rating system for emissions, known as the Japanese Industrial Standards (JIS) and Japanese Agricultural Standards (JAS), which apply to formaldehyde-emitting building materials [41]. The Consumer Product Safety Commission (CPSC) has publically stated that free formaldehyde levels should generally be below 0.03 ppm in closed environments [42].

Today, although many international organizations agree that formaldehyde exposure must be controlled and governed, each provides a different value for controlling its levels in the workplace and at home. The American Conference of Governmental Industrial Hygienists (ACGIH) commends a threshold limit value (TLV) of 0.3 ppm as an 8 h time-weighted average (TWA) [43]. Globally, the WHO recommendation is 0.1 mg/m^3^ (0.08 ppm) for the general indoor exposure limit [44]. Several Member states of the EU have already set national Occupational Exposure Limits (OEL) to free formaldehyde. However, Europeans are placing more restrictions on formaldehyde emissions to reach a zero-emission limit effectively. For the protection of workers, Formacare decided in 2019 to establish a voluntary agreement to apply an OEL for formaldehyde. The limit was set at 0.3 ppm for the 8 h TWA (Table 1), the pan-European maximum exposure recommended by the European Scientific Committee on Occupational Exposure Limits (SCOEL). This suggested value was approved by EU policy makers to be the new EU Binding Occupational Exposure Limit (BOEL), which took effect on 11 July 2021 [45].

In addition, finding natural raw materials as alternative resources is the only solution for the upcoming shortage of petroleum resources.

### 2.3. Current Biobased Wood Adhesives

**Figure 1:**
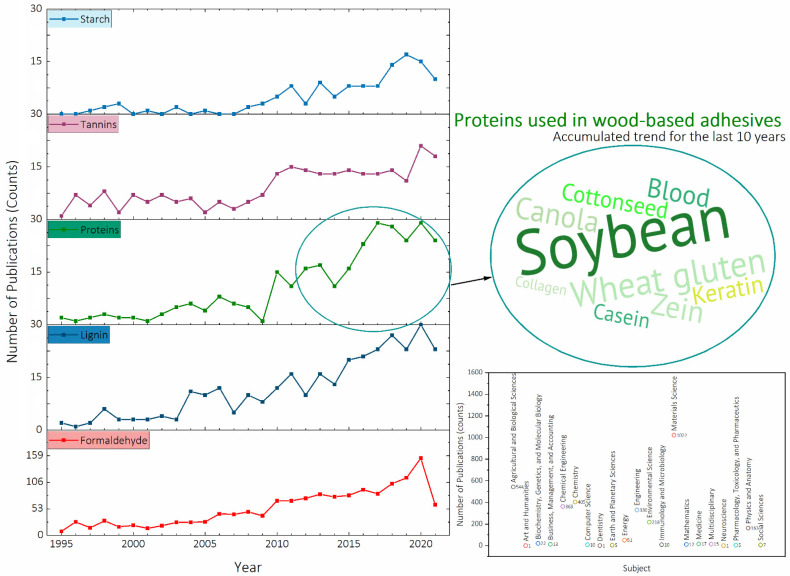
Distribution of the number of publications indexed in the Scopus database.

Biobased products are made from natural raw materials, which do not come from mineral or fossil sources. Specifically, the term “biobased adhesive” is now used in a clearly defined and narrow meaning, involving materials exclusively from a natural, non-mineral source that can be used and modified to obtain the best properties required to substitute synthetic adhesives [49]. However, even if some adhesives are biobased, attention must be paid to their manufacture and use, which could lead to significant energy input and emission of organic pollutants [50]. As a result, the qualification “biobased” is no longer sufficient, and the term “sustainability,” which refers to their environmental footprint and cost, should be included over their entire life-cycle. One way of approaching the sustainable concept might be to increase the natural binders of the adhesive systems, leading to a significant development of environmentally friendly wood adhesives from renewable resources. Different biomass resources such as lignin [51,52,53,54], starch [1,55], tannins [56,57,58,59], vegetable oils [60], and proteins [21,61,62,63] are among some of the latest reported studies (Figure 1). The latter are the most abundant class of macromolecules within biobased materials as they constitute the main organic building blocks in living organisms. Whether of animal or plant origin, proteins represent abundant, renewable, inexpensive, and eco-friendly biomass resources that can be employed as raw materials for new biobased industrial products. Many vegetable proteins have been adopted to develop wood adhesives, such as wheat gluten, zein, canola, cottonseed protein, and soy protein and their blends in addition to the animal-based proteins including feather keratin, casein, spider silk protein, bone protein, blood meal, leather cysteine, among others. Protein-based adhesives for engineered wood panels will be discussed in more detail later in this review.

## 3. Mechanics behind Bioadhesive Development

### 3.1. Mechanism of Adhesion

Adhesion science is a multidisciplinary topic that includes surface chemistry and physics. It is defined as the interatomic and intermolecular interaction at the interface of two surfaces [64]. Attractive forces can occur when two materials come into contact, depending on the chemical nature of the surfaces of the materials concerned: physical or chemical adsorption (chemisorption). If the interaction between the adhesive and the adherend is weak, these materials will come loose if a force is applied. Explaining the adhesion mechanism in simple terms is difficult due to the complexity and evolving interpretations of the subject [65]. Therefore, adhesion mechanisms have been investigated to be reliant on the surface characteristics of the materials. The eventual aim is to identify a single mechanism that explains the adhesion phenomena [64,66,67,68,69,70,71]. In general, it is approved that there are three primary mechanisms of adhesion: (I) mechanical interlocking, (II) physical bonding, and (III) chemical bonding [72]. All these mechanisms are responsible for bonding, and typically, one of them plays a dominant role.

Mechanical interlocking is probably the principal mechanism by which adhesives adhere to porous structures, such as wood. It depends on the size and the geometry of the locking sites; thus, surface topography. Further diffusion into the fine microstructure increases the surface contact area between adhesive and wood for stronger mechanical interlocking. The most lasting structural bonds to wood are believed to improve when an adhesive penetrates deeply into cell cavities and diffuses into cell walls to make molecular-level contact with the hemicelluloses and cellulosic compounds of wood. If an adhesive penetrates deeply enough into sound wood and becomes stiff enough after curing, the strength of the bond can be likely expected to surpass the strength of the wood [73].

Physical bonding consists of three intermolecular attraction forces essential to form bonds between the molecular structures of wood and adhesive polymers. Generally, these are called van der Waal’s forces, London forces, and hydrogen bonding. Hydrogen bonding is essential in the interfacial attraction of polar adhesive polymers, hemicelluloses, and celluloses, rich in polar hydroxyl groups. These physical forces of attraction are sometimes indicated as specific adhesion, which is particularly important in wetting and adsorption of adhesive polymers onto the molecular structures of wood.

Covalent chemical bonds form when non-metallic atoms share electrons to form molecules. They are strong, high-energy bonds and present more than 11 times the strength of the hydrogen bond. However, the assumption that the most robust interaction will control the adhesion is insufficient since the latter is the product of the strength of each interaction times the frequency of its occurrence. Thus, covalent bonds that happen only rarely may not influence bond strength as much as the more common hydrogen bonds or dipole–dipole interactions [74].

### 3.2. Wood Bonding and Adhesion Theories

Exploring the theories responsible for wood adhesive bonding has been an essential aspect of wood science technology in recent decades, alongside the development of wood-based composites, with gluing of wood representing more than 80 percent of its applications [75]. Thus, studying adhesion theories requires understanding of the characteristics of wood as a material, surface science, polymer characteristics, and the interactions between polymers and surfaces [76].

Wood has a unique structure, different from other materials since it is natural, heterogeneous, and exhibits variability in species, cut-off, and specific specimen [77]. Therefore, adhesion to wood most often relies on physical bonding and mechanical interlocking. In addition, because of the intrinsic porosity of the wood, the adhesive generally penetrates deeply into several cells for excellent mechanical interlocking. Still, the most durable bonds to wood occur when the glue diffuses into the cell walls to create hydrogen bonds with the hemicelluloses and cellulosic compounds [78].

**Figure 2:**
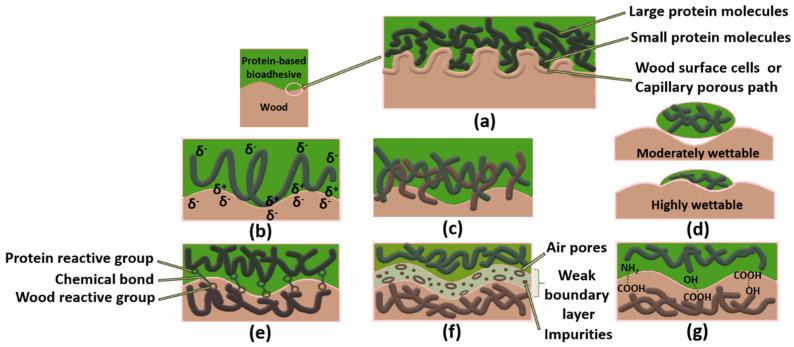
Adhesion theories in protein–wood adhesive bonding, mechanical interlocking (**a**), electrostatic (**b**), diffusion (**c**), wettability (**d**), chemical (**e**), weak boundary layer (**f**), acid-base (**g**). (Adapted from: [81,82]).

The most recent state of the art classes adhesion theories into seven models or fields. These are mechanical interlocking, electronic or electrostatic theory, diffusion theory, adsorption (thermodynamic) or wetting theory, chemical (covalent) bonding theory, theory of weak boundary layers, and acid-base theory [76,79,80]. The last four are based on adsorption/surface reaction. The seven adhesion theories are summarized in Figure 2.

Even if, usually, each phenomenon contributes to the whole strength of adhesion, in the case of wood, the primary adhesion mechanism includes interfacial secondary interactions such as van der Waal’s forces, dipole–dipole interactions, and H-bonding [83,84]. This implies that the two main theories involved in the case of wood bonding are generally the adsorption and acid-base theories. Although several researchers doubt the presence of covalent bonds with wood [82,83], some studies associate the adhesive strength of protein-based systems with the existence of covalent linkages between the functional groups of the adhesive and those of wood [84,85,86]. This hypothesis is reasonable with some adhesives, especially when the formulations contain functional groups such as azetidine groups [87] and isocyanates [84,85], which can react with wood during curing conditions.

Moreover, due to the porous structure of wood, it is generally accepted that some penetration of the adhesive from the surface leads to strong mechanical interlocking, advantageously providing stronger bonding [82,83]. Therefore, under specific curing conditions, the combination of physical bonding and mechanical interlocking can further contribute to the overall bond strength in wood adhesion with protein-based adhesives. Readers who wish to gain an in-depth understanding of the interaction forces, adhesion mechanism, and thermodynamics of adhesion are recommended to consult the references: [88,89]. To help understand the difference between the theories and mechanisms of adhesion, the possible mechanisms underlying each of these theories are schematically represented in Table 2.

### 3.3. Wood Adhesion Considerations

Since adhesive bonds rely on interactions at the interface, the physical and chemical characteristics of the surfaces are crucial for adequate joint performance. Even if wood surfaces appear smooth and featureless, they become peaks, valleys, and crevices, littered with loose fibers and other debris at the microscopic scale. Such surface conditions lead to gas pockets and blockages that prevent complete wetting by the adhesive and introduce stress concentrations when the adhesive has cured. Thus, wood surfaces should be flat, smooth, and free of machine marks and other surface irregularities, including burnishes, oils, dirt, planer skips, torn and chipped grain. Properly planed, flat surfaces ensure that an adhesive layer of uniform thickness can be homogeneously spread over the adherend so that under pressure, the adhesive will flow into a uniformly thin layer [73].

Moreover, for two wood adherents to be joined with maximum power, a liquid adhesive should be wet and spread readily to make intimate contact with both surfaces. Molecules of the adhesive must diffuse over and into each surface to contact the molecular structure of the wood. Thereby, the wettability of the surface is strongly correlated to the adhesion. As most surface modifications will influence both the chemical composition and the roughness of the surface, the separation of these two is important for the overall understanding of the adhesion process.

As a cellular structure, wood is porous and reveals different porosity levels, depending on species. Porosity can be advantageous in some cases but also deleterious in others. Flow into the wood allows for more mechanical interlock and bonding area than most substrates. However, this may allow adhesive to flow away from the glue line, leading to either a starved glue line or excess consumption of adhesives [90].

Water exists naturally in living trees, mainly as free water in cell lumens and adsorbed water within the cell walls. The entire water content of wood can vary well above 200% (based on oven-dry weight). However, once the free water is eliminated from cell lumens by drying, approximately 30% remains bound within the cell walls. Water has a persistent molecular attraction to wood, mainly through hydrogen bonding with the hydroxyl groups of cellulosic compounds. Thus, the cell walls remain saturated with moisture (called the fiber saturation point) until the moisture content of the surrounding air falls below that of the saturated cell walls. The actual moisture content at fiber saturation (roughly 30%) varies, depending on species, tree, temperature, and pressure. This is the critical point where the wood starts to shrink. Due to moisture content variation, dimensional modifications have a broad-ranging and significant impact on the joints’ final strength and durability, development of surface checks in the wood, and the dimensional stability of the bonded assembly. Substantial changes in moisture content from that at the time of bonding will cause shrinking or swelling stresses that can produce warping, twisting, and surface irregularities and thus significantly weaken both wood and joints. As a result, wood should not be bonded with a high moisture content, especially high-density hardwoods with wide shrinkage coefficients, unless the moisture content in service is also assumed to be high. The wood must be sufficiently dry so that even if moisture is increased during bonding, the moisture content is at about the level expected for the assembly in service.

Pressure during bonding provides several practical purposes: it forces entrapped air out of the joint, leads to molecular contact between adhesives and wood surfaces, causes adhesive to diffuse into the wood structure for more effective mechanical interlocking, squeezes the adhesive into a continuous thin film, and maintains the assembly in position while the adhesive cures. On the other hand, if pressure is too high, the adhesive can over-penetrate porous wood and cause starved joints deficient in bond strength. The most substantial joint outcome is when the coherence of the adhesive permits the use of reasonably high pressures that are consistent with the pressures recommended for the density of the wood.

## 4. Protein-Based Adhesives for Wood-Based Composites

Since it is difficult to determine the adhesive properties of proteins without a thorough knowledge of their structural complexity, involving specific hierarchical orders, a brief description of their structure and fundamental properties is proposed hereafter.

### 4.1. The Shape and Structure of Proteins

Proteins are the most frequent class of macromolecules within the different biopolymers and act as the main organic components in living organisms. Approximately 50 percent of the cell dry weight consists of proteins [31].

Proteins are biological polypeptide macromolecules whose backbone is a sequence of amino acids (AA) linked linearly by peptide bonds, i.e., condensation reactions between their carboxylic acid and amino groups, called the primary structure [91,92]. Considering that some proteins are composed of up to about 30,000 AA selected from one of the 20 available (+2 in some specific cases), a wide variety of sequences can be formed, depending on the AA’s number, percentage, and chaining. The latter has at least one carboxyl group and an amino group, making them ampholytic, since they can react as either acid or base, depending on their environment. This aspect typically signifies that pH control is unnecessary, mixing the amino acid with a wide range of additives [21]. Furthermore, the side chain of AA can be aliphatic, aromatic, or heterocyclic, with many types of functional groups leading to a broad diversity of interactions between hydrophobic and hydrophilic (acidic, basic, hydroxyl, amino, thiol, among others) side groups.

The primary structure determines the secondary, corresponding to a protein folding, induced by local interactions between stretches of a polypeptide chain and includes α-helix and β-pleated sheet structures. This fundamental structure is controlled by interactions between side-chain functional groups, including covalent disulfide bonds, electrostatic and hydrophobic interactions, and hydrogen bonding. Then the three-dimensional tertiary form where α-helix and β-pleated sheets fold into dense globules, corresponding to the overall shape of the protein, is called the tertiary structure. Proteins can be divided according to their tertiary structure, depending upon their functions: globular or fibrous. Globular proteins have a spherical-like structure and are one of the most abundant proteins. They are soluble in water and form colloids. Fibrous or linear proteins are made up of regular, long, and narrow amino acid strands and are not soluble in water [93]. This complex structure gives the proteins physical and chemical properties and controls their essential functions. The interactions between the surfaces of these globules lead to the quaternary structure, i.e., complexes through various types of non-covalent bonds between different proteins (different polypeptide chains) [94].

It is important to remember that most natural sources contain various proteins, and in many cases, proteins are thoroughly associated with other materials, such as carbohydrates, affecting their properties. Thus, many methods have evolved to analyze proteins and measure their properties [95]. Although the discussion of proteins ends, at times, at the quaternary structure, there are often higher-order macromolecular structures, coming from larger protein agglomerates [96] or by association with carbohydrates [95]. This process becomes significant during the aqueous gelation process in water, linking the proteins to form a complex macromolecular structure [61].

### 4.2. Protein-Based Adhesives: General Approach

Two main reasons can explain the development of protein adhesives: firstly, it allows the production of materials with specific physicochemical properties that make them relevant for industrial utilization; secondly, it replaces synthetic compounds with natural resources, bringing responses to concerns about health hazards from the synthetic monomers and the carbon footprint of the material. Moreover, they can be processed similarly to synthetic polymers, with the advantage of sharing wood biodegradability (for uniform end-of-life management) and generating a smaller carbon footprint.

For application as adhesives, critical physicochemical properties have to be controlled: good storage stability, flowability, wetting ability, and stickiness of the protein dispersions and solutions, as well as strength and resistance to deterioration under the different environmental conditions of the resulting adhesive bonding [82,97]. With the evolution of science, it has been possible to modulate the properties of proteins and establish characteristics for specific applications by controlling the processing conditions or by chemical modifications.

Even if linear proteins can form spatial networks by cross-linking more efficiently, most plant proteins exhibit a globular structure, from which wood adhesives can be prepared with good adhering performance [98,99,100]. A functional morphology for globular proteins is a string of beads with branch points to give a three-dimensional gel that traps a lot of water [58,61]. These larger aggregates probably supply the bonding strength for protein adhesives.

There are four main strategies for formulating wood adhesives using globular proteins. The first method involves the dissolution of proteins in a proper solvent to make them easier to process, using various physical and chemical treatments. This method allows more exposed specific functional groups of a protein, such as polar and non-polar groups [5], making them more compliant to interactions with the substrate. Another way is the denaturation of proteins through different ways: exposure to heat, ultraviolet (UV) radiation, acid/alkali, organic solvents, denaturing agents, such as guanidine chloride, and urea, are the most commonly used to accomplish this [61,101,102]. A third approach involves improving the entanglement of polypeptide chains, which can enhance material strength by increasing cohesive strength. Finally, crosslinking polypeptide chains after curing at high temperatures and high pressure reinforces bonding strength and water resistance [100,103]. This method requires the exposure of reactive functional groups such as carboxyl, amino, hydroxyl, and sulfonamide groups, which crosslink the polypeptide chains.

An overview of the most relevant protein biobased adhesives that can be used in the wood industry is proposed hereafter, providing a guide across the spectrum of plant and animal adhesives to match specific needs and opportunities.

### 4.3. Adhesives from Plant Proteins

Proteinous feedstock resources for adhesive development can be obtained from various resources. Previously, plant-based adhesives occupied the larger part of the market, owing to their abundant availability as raw materials and the low cost of processing. These have now been replaced with synthetic resins, which offer superior adhesive strength and water resistance. However, these challenges can be met using the unique properties of each of these renewable proteinous materials.

#### 4.3.1. Wheat Gluten

Wheat gluten is an attractive raw material for sustainable wood adhesives as a cohesive, viscoelastic proteinous by-product obtained from wheat starch processing. It is a complex combination of 80% wheat protein, with the rest being lipids, polysaccharides, and minerals. Wheat gluten comprises four main protein groups, albumins, globulins, gliadins, and glutenins, the latter two accounting for 85% of the total protein content [104]. Albumins and globulins are water and salt soluble, while gliadins are soluble in alcohol, and glutenins are dispersible in dilute acids or bases. Gliadins include distinct polypeptide chains, basically connected via hydrogen bonds and hydrophobic interactions, while glutenins are composed of polypeptide chains linked together by interchain disulfide bonds. In addition, hydrogen bonds between repeated regions of high molecular weight glutenin subunits lead to the elastic properties of wheat gluten. For their part, gliadins contribute to viscous properties due to their globular structure.

The bonding properties of gliadins have lesser water resistance than glutenins because of their overpenetration into wood materials [105]. Some studies have been reported to improve the adhesive properties of wheat protein, including sodium hydroxide treatment to enhance the thermal stability of reed particleboard [106], or alkaline and enzymatic hydrolysis to form smaller peptides, improving dry bonding strength and wet bonding strength after more extended treatment. Other modification methods involving enzymatic hydrolysis and heat treatment were proposed, successfully enhancing the water resistance and bonding strength of bonded beech wood specimens [107]. The effect of denaturing proteins to unfold them and improve the solubility has also been investigated, keeping in mind that the adhesive performance of proteins is influenced by their dispersing properties and their ability to interact with the wooden substrate [107]. Other research has demonstrated the effect of adding crosslinking agents, whereby selecting the appropriate dispersing agent with a suitable crosslinker can enhance the board properties [108]. Further works have proposed eco-friendly binders in the form of thick spent sulfite liquor (TSSL) combined with wheat flour to produce fully biobased particleboards with appropriate mechanical performance [109].

#### 4.3.2. Soy Protein

Soy is the most well-known protein used to develop biobased adhesives, being beneficial in terms of availability, biodegradability, and cost. Soybeans are used abundantly in biodiesel, biocomposites, dyes, and wood adhesives in different engineered particleboards [110]. Soy proteins are made up of 10% albumins and 90% globulins, and as mentioned earlier, the former can be extracted by water, while the latter by moderate salt solutions [111]. Soy proteins are globular and possess a more hydrophilic character than wheat gluten.

Even if a large amount of research has been conducted on soy protein for wood applications, soy protein-based adhesives have been limited due to poor water resistance. Therefore, research to change their molecular conformations using modifying agents has been well recorded in the literature to counter this limitation. The methods include enzymatic modification [112], chemical denaturation [112], alkaline treatment [113], crosslinking [114], and addition of additives [115], like urea [116], and sodium dodecyl sulfate [117]. These chemical modifications applied to soy protein are based on reactions associated with amido and amino groups. Another approach that can be applied is to mix soy protein products with other natural materials such as lignin and tannin [115,118].

#### 4.3.3. Cotton Protein

Cotton is a non-food industrial crop and is mainly developed for its fiber. However, cottonseed protein is currently being studied as a potential adhesive for wood bonding like other plant proteins. Thus, it represents a valuable agro-industry biobased raw material with numerous applications due to its chemical composition (fibers, proteins, carbohydrates, and lipids) [119]. In terms of amino acid composition, it has nearly the same primary amino acid components as soy protein, those with the highest levels being arginine, glutamic acid, and glutamine [120].

Over the years, there has been a recurring interest in improving the properties of cottonseed protein as adhesive. In the 1950s, the ability to apply cottonseed protein in gluing formulations was explored, leading to acceptable adhesives relative to casein and peanut meal [121,122,123]. The greater adhesive strength of cottonseed protein has been demonstrated by comparing it to soy proteins when tested on maple wood veneer [87]. Additional studies have been done to improve the water resistance of cottonseed protein by adding modifiers. For example, a wide variety of cationic and anionic additives, including amino acids, fatty acids, and small molecules, have been used to compare cottonseed and soy protein-based adhesives performances regarding their bonding strength with different wood veneers. Performance was superior for the cottonseed protein in the presence of anionically charged molecules such as aspartate, glutamate, acetate, adipate, and butyrate. At the same time, no improvement was identified in the case of soy protein. Moreover, the bonding of cottonseed protein-based adhesive on different wood veneers was far more potent than soy protein-based adhesives on the same veneer [124].

#### 4.3.4. Rapeseed Protein

Other plant proteins have not been as commonly used as soy or used commercially before, perhaps due to limited availability and cost. Rapeseed is rich in oil (30–45%) and protein (20–30%). However, because of the significant advances in all plant production levels, plant proteins are considered the least expensive compared to other protein sources. Despite the availability of rapeseed in various areas, its toxic nature has limited its use as an agricultural product [125,126]. On the other hand, these toxic components do not affect proteins, which means that the food market’s limitations are not applicable for adhesives. Numerous extraction techniques have been used to extract the protein part from oilseeds. For instance, the extraction is feasible with water or alkali, NaCl, and sodium hexametaphosphate solutions. The most commonly used method is alkaline extraction due to it having the highest yields [126]. Isolation of the protein is carried out by adding enough water-miscible solvent, such as ethanol, to an aqueous solution containing protein extracted from the meal, whereby the proteins present precipitate.

Some research has focused on the influence of chemical modification on the adhesion performance of rapeseed protein to produce wood adhesives. For example, sodium dodecyl sulfate, CaCO_3_, ZnSO_4_, CaCl_2_, and octenyl succinic anhydride were tested as chemical modifiers, alone or in combination. Based on chemical and concentration, modifying canola protein enhanced the shear strength of canola protein wood adhesives. Of the chemical modifications used, the 3.5% OSA modification had wet, dry, and soak shear strengths greater than unmodified canola protein, making it the most successful chemical modification [127]. Besides, there are still some challenges to overcome in terms of adhesion, mechanical properties, and moisture sensitivity of these proteins in non-food applications [128].

#### 4.3.5. Zein Protein

Zein is another class of plant proteins called prolamin proteins, referring to the high proline amino acid content. It is the major storage protein of corn and comprises around 45–50% of the protein in corn [129]. However, Zein isolate is not used directly for human consumption because of its negative nitrogen balance and poor solubility in water.

The high proportion of non-polar amino acid residues and deficiency in basic and acid amino acids is responsible for its hydrophobic character. For this reason, zein was chosen as one of the primary ingredients that can be used to prepare protein-based adhesives. Previous work reported an easy strategy for manufacturing a zein-based adhesive induced by chelation with metal cations [130], using different metal chloride solutions. It was found that the processing with five wt.% FeCl_3_ aqueous solutions (Fe(III)@zein/SDS adhesive) conduced to the best performance, ensuring good adhesive strength and water resistance. Moreover, the reported method did not require any complicated process for its synthesis or modification.

### 4.4. Adhesives from Animal Proteins

Efficient utilization of non-edible animal by-products is essential for environmental protection and sustainability. Most animal by-products are not suitable for human consumption because of their unusual physical and chemical characteristics. As a result, a precious source of industrial proteins is undervalued, and the cost of disposing of these products increases. For this reason, up to 13% of the current bioadhesives market is covered by animal-based ones [131]. The protein from non-edible animal by-products is likely to develop as a sustainable protein resource for different industrial applications.

#### 4.4.1. Casein Protein

Casein is the main protein of milk; it has a micellar structure, which is made up of 94% casein protein (α-,β-, and κ-casein) and 6% colloidal phosphate calcium (CCP) [132,133]. The casein micelle is a complex polymer aggregate that interacts via hydrophobic and electrostatic interactions and calcium bridging [63]. It is highly heat-stable due to the κ-casein layers [134]. It can be precipitated by adjusting the pH of skim milk to 4.6 with mineral acids like hydrochloric or sulfuric acid or by acidification due to the lactic acid produced in situ by bacteria [135]. Otherwise, coagulation can also be obtained by a complex set of an enzyme called rennet [95].

Casein glues have been used since ancient Egypt [136]; they aroused great interest due to their better color and remarkable water resistance compared to other animal glues [137]. The adhesive is fabricated using casein and an alkali component (e.g., hydrated lime or sodium hydroxide) in modest proportions [138,139,140,141]. Most casein-based adhesives are found as a powder, while water is added at the time of use [136]; adjusting the lime or sodium hydroxide proportions can produce adhesives with different properties. For instance, a recent study tested different formulations containing casein powder, water, lime, sodium silicate in various glue amounts to investigate the performance of casein-based adhesives for bonding ash veneers. The highest efficiency of mechanical and physical properties was recorded for the samples glued with caseins and an increased amount of lime [62].

#### 4.4.2. Blood Protein

From blood, which is a by-product of slaughterhouse operations, dried soluble blood powder is produced generally by low-temperature methods such as spray or vacuum dryer. Animal blood flour is made of 80–90% proteins, globular in type.

Blood-based adhesives are manufactured from whole blood. As with casein adhesives, blood-based ones are formulated by redissolving the blood in the water and dispersing it with an alkaline compound, such as caustic soda or hydrated lime, necessary for protein unfolding and attaining an aqueous dispersion with suitable viscosity. In addition to providing an alkaline environment, sodium silicate could act as a curing agent [142]. In the dry state, blood-glues exhibit less strength than casein glues, but they resist moisture much better [143]. Until synthetic resins became generally available, blood-glues were mainly used to enhance the water-resistance properties of other protein adhesives. For instance, the casein-blood adhesives were considered the most durable for exterior PW until PF adhesives were eventually formulated to provide a better adhesive [144], [125]. Moreover, the higher protein content and hydrophobic amino acid content of blood are beneficial for fabricating superior adhesives [145].

#### 4.4.3. Keratin Protein

Keratin is a member of a fibrous structural proteins family. It is the main structural material making up hair, nails, horns, hooves, wool, and feathers. Based on the auxiliary order, keratins can be α-keratins which are found in all vertebrates, or β-keratins existing in birds and reptiles [146,147]. Keratins do not act like other proteins and are usually not solubilized by ordinary methods. However, under appropriate conditions, especially at low pH and through reducing/oxidizing agents, they become more water-soluble and chemically reactive due to disulfide, amino, and carboxylic acid moieties [148].

Studies have been conducted on feathers, being the main by-product of the poultry industry, keratin has several advantages over soy proteins in the case of wood adhesives: (1) its hydrophobic nature provides better water resistance; (2) keratin is a fibrous protein, which is easier to hydrolyze than globular ones such as soy; (3) it possesses natural anti-mildew components. Recently, novel methods for developing low-cost biobased formaldehyde-free adhesives based on chicken keratin feather and soybean meal (SM) could improve the fluidity and wettability of the protein-based adhesive compared to SM-only adhesive [149,150]. Furthermore, studies on keratin and collagen extracts for the formulation of wood adhesives showed antioxidant activity and a considerable reduction in free formaldehyde emissions from urea-formaldehyde adhesives [151,152].

#### 4.4.4. Collagen Protein

Collagen, the highest abundant structural protein, has a broad amino acid composition strictly different from other proteins and has been proved to have a unique triple-helical structure [153]. The collagen protein forms elongated triple-stranded helical coils rather than individual protein chains being coiled globules [154]. These coiled proteins that make up the collagen fibrils give good strength and good flexibility. The high contents of proline and hydroxyproline inhibit the protein chain from forming a globular shape, and the many polar groups provide good interaction between the chains. There is huge variability in the collagen due to the capability to use the collagenous waste from different mammals [155], consequently based on its source (skins, connective tissue, cartilage, and bones) and the age and type of animal source. Generally, the waste is treated using various procedures because of the need to separate the collagen from other materials. After cleaning to remove most of the inorganic and other organic molecules, the fibrils are disentangled and hydrolyzed using an inorganic acid and heat [125].

The origin of the collagen and the preparation method determine the physical, chemical, and mechanical properties of the adhesive. However, the difference in source becomes less crucial since the chains are shortened to lower the molecular weight of coiled proteins [154]. Unless the addition of tanning agents has altered them, which drives them to become relatively water-resistant, collagen-based adhesives usually swell when exposed to water and redissolve when heated, even after centuries [155]. Wooden joints glued with fish adhesive or cold liquid hide glue have been demonstrated to dissociate with water following six months of natural aging or relative humidity and temperature cycling [156]. The resolubility of animal adhesives may be decreased in cases where the protein has encountered metal ions (e.g., metal foils, tools, pigments) or with certain organic pigments and tannins, either before, during, or even after their application [157,158]. The resolubility of collagen-based adhesive with no additives is dependent on the environment of exposure rather than being predetermined by the type of glue [159]. Despite the lack of recent studies on collagen as wood adhesive, it has been used widely in ancient times. A study was conducted on a bone sculpture-inlaid wooden artifact in the Xiaohe Cemetery in China, where the adhesive was identified as bovine-specific peptides of collagen type I [160]. Not long ago, a novel bioadhesive was easily prepared using collagen hydrolysate extracted from leather waste as the raw material. The low-cost glue displayed a properly crosslinked three-dimensional structural network, enabling good interactions with wood, thus demonstrating pretty good adhesive strength and water resistance [161]. The advantages and disadvantages of each of these proteins regarding their performance are summarized in Table 3.

## 5. Future Opportunities for Protein-Based Adhesives

Protein-based materials are increasing in their attention for use as adhesives due to their independence from petroleum resources and their beneficial environmental footprint. In the long term, protein-based materials can provide adhesives compared in their properties to their formaldehyde-based counterparts beyond their renewability.

### 5.1. Modification of Protein Adhesion

An exceptional superiority of protein-based adhesives is the dependence of their adhesive strength on their different levels of structure. Proteins can be processed similarly to synthetic adhesives. For instance, folding the protein chains can be induced by adding crosslinking agents or compounds that can interact with the chains. This was demonstrated in the previous example in comparing cottonseed and soy protein as adhesives for maple veneer [87]. Other mechanisms by which proteins can be affected are hydrolysis [172], pH change [173], and thermal treatment [174]. Therefore, simple measures can switch the protein properties according to the desired application. In general, the highest adhesive strength for proteins is noticed near their isoelectric point, in which their solubility is lowered, increasing the interaction between protein chains and their hydrophobicity [175,176], thereby, adjusting the water-resistance of protein adhesives.

### 5.2. Reduced Human and Environmental Toxicity

The major driving factor for the research into alternative adhesives for wood composite materials is avoiding the harmful emissions of formaldehyde-based adhesives. Another factor for introducing biobased materials has been their favorable impact on climate change. Generally, biobased materials do not lead to the consumption of fossil fuels. Moreover, their environmental benefits go beyond these considerations to their relative toxicity to humans and biodegradability. Lastly, solutions that can be developed are less likely to contain harmful and toxic chemicals and perhaps are more likely to be synthesized in line with green chemistry principles.

### 5.3. Lower Quantities of Adhesive and Avoidance of Organic Solvents

Environmental profits are fulfilled when the toxic substances are discarded from the adhesive formulation and when the total amount of adhesive required decreases. This can reduce the overall energy needed for adhesive production. In some instances, biobased adhesives, particularly proteinous, can achieve the same results as petroleum-based adhesives even if applied in lower amounts [177]. Furthermore, the water solubility of specific proteins, such as whey protein used to make an aqueous adhesive for glue-laminated timber, makes the use of emulsifying agents unnecessary [85].

### 5.4. Commercially Relevant Advantages

Protein-based adhesives can provide commercially relevant benefits. The most valuable one is pricing. For instance, keratin-waste animal protein can be obtained at a low cost. Another example was the hydrolyzed soy flour in an alkaline medium in the presence of phenol and integrated into a phenol-formaldehyde resin to produce an oriental strand board [178]. According to the estimated prices of soy flour and phenol, the authors approximate a 30% replacement of the phenol, resulting in a 20% material cost-saving while maintaining comparable performance.

## 6. Perspectives

One of the weaknesses of proteins is their sensitivity to water, meaning hydrolytic degradation. Different measures can counteract this, including increasing the protein adhesive crosslinking density through crosslinking agents and hydrophobic groups. Examples of this are the above methods used to modify protein adhesion by altering their structure and exposing the hydrophobic groups. Other ways could be simply to incorporate the more hydrophobic proteins such as keratin, zein, and blood proteins that can enhance the water-resistance properties of other protein adhesives.

## 7. Conclusions

This article reviews the recent developments of plant and animal proteins as promising candidates to formulate wood adhesives. Protein based-adhesives offer a sustainable solution to indoor air quality and formaldehyde exposure concerns. All the raw materials discussed above are renewable and available. They can substantially reduce emissions (formaldehyde and VOC) when substituting synthetic adhesives currently used in the wood industry, while not being an application competing with human or animal nutrition, but instead emerging from such industries as a by-product. On the other hand, protein-based adhesives have drawbacks that hinder their industrial use, mainly poor water resistance for hydroxyl group-rich materials and viscosity for long molecule chain polymers. Developing an environmentally friendly wood adhesive system that is competitive with urea-formaldehyde and phenol-formaldehyde resins is feasible to achieve by a combination of technologies, such as protein denaturation, followed by chemical modification of the denatured protein and chemical crosslinking. However, challenges are yet to be addressed regarding the costly chemical modifications, which hopefully can be regulated by incorporating hydrophobic proteins for better water resistance performance.

## Figures and Tables

**Table 1:** Occupational Exposure Limits (OELs) for formaldehyde in European countries ([17,46,47,48]).

Countries	TWA (8 h)		
	2006	2015	2021
	ppm	ppm	ppm
Austria	0.3	0.5	0.3
Belgium	0.3		
Denmark	0.3	0.3	0.3
Finland	0.3	0.3	0.3
France	0.5	0.5	0.3
Germany	0.3	0.3	0.3
Greece	2		
Ireland	2	2	0.3
Italy	0.3		
Netherlands	1		

**Table 2:** Adhesion theories and possible mechanisms.

Theory	Mechanism	Strength of Interaction
Mechanical interlock	Mechanical forces	Variable
Electrostatic	Ion–dipole interactions	Strong
Diffusion	Interdiffusion	Variable
Adsorption/wettability	Van der Waals, dipole-dipole interactions	Weak, moderate to strong
Chemical bonding	Covalent bond	Very strong
Weak boundary layer	Defects at interface	Variable
Acid-base	H-bonding, dipole–dipole, or ionic interactions	Moderate to strong; very strong

**Table 3:** Advantages and disadvantages of various protein-based wood adhesives.

Source	Proteins	Advantages	Disadvantages	References
Plants	Wheat Gluten	Dispersible in alkali and acid High amount of hydrophobic amino-acids Abundant Cheapest protein source	Water-insoluble Poor shear strength Highly viscous due to swelling of starch by water absorption and release of amylose chains	[25,107,162]
	Soy meal	Abundant Inexpensive Good strength under drying conditions Cold curing High protein content Good thermal resistance	Limited water resistance Poor wettability High viscosity Long hot-pressing time Sensitive to microbial degradation Not suitable for exterior applications	[6,25,114,163]
	Cottonseed	Non-food crop Superior adhesive strength Hot water resistance compared to soy protein	Costly extraction of protein Poor water resistance	[6,120]
	Canola	Abundant oilseed crop Non-food crop Chemical resistance to hot water	Needs a lot of chemical modifications	[127,164,165]
	Zein	Hydrophobic protein Water-resistant Good adhesive strength Low-cost preparation A lot of chemical modifications is unneeded	Yellow color due to xanthophylls, carotenoids, and other color pigments present in corn Relatively high cost of extraction due to organic solvents needed	[129,165]
Animals	Casein	Strong joints that are largely resistant to water Relatively safe to work with Moderately high dry strength Moderate resistance to water	Take a long time to set Quite susceptible to degradation by fungi and other organisms Relatively expensive due to preservatives Eight gallons of skim milk are required to make one pound of dry casein Short pot life Not suitable for exterior uses	[166,167]
	Blood	Very rapid setting with heat Moderate to high dry shear strength Moderate to high water resistance Moderate resistance to microorganisms Does not stain wood Can be applied using both hot and cold presses Easy to handle because of its relatively low viscosity	Produce dark glue lines Blood drying is an energy-intensive process Blood meal (BM) is inclined to agglomerate, which is adverse to bonding About half of the amino acid of BM is non-polar, resulting in low bonding capacity The connection of BM protein molecules is mainly hydrogen bond, which leads to bad water resistance of resultant adhesive in the absence of crosslinking agents	[25,145,168]
	Keratin	The most abundant among animal sources Cost-effective Hydrophobic Broad chemical tool-set and structural variation Effective filler for composite polymers Available thiol moiety Water resistant Fungal decay protection Appreciable adhesive strength under dry conditions	Non-homogeneous composition Poor solubility and thus unusual extraction processes needed Disinfecting process is needed to apply them	[169,170]
	Collagen	Low risk of infection Highly non-polar Globular in nature that minimizes water interaction	Needs processing to separate the collagen from other materials Moisture sensitivity	[171]

## Data Availability

Not applicable.
